# Supplementary material for: Using allocative efficiency analysis to inform health benefits package design for progressing towards Universal Health Coverage: Proof-of-concept studies in countries seeking decision support
Source: PLoS One. 2021 Nov 29;16(11):e0260247. doi: 10.1371/journal.pone.0260247 (PMC8629222; doi:10.1371/journal.pone.0260247)
Supplement: S1 Appendix — (PDF) [file pone.0260247.s001.pdf]

Supplement 1: Outline of HIPtool

Background

This document briefly outlines the Health Interventions Prioritization Tool (HIPtool), which combines all available country-specific evidence on intervention cost, coverage, and impact with demographics, disease burden, resource availability, and other data. HIPtool can help inform policymakers via the three aims of determining which sets of interventions: (a) maximise disability-adjusted life years (DALYs) averted, (b) maximise equity, and/or (c) maximise financial risk protection. The results from these analyses can then be linked to platforms for delivery to provide an initial step towards developing an optimal HBP.

Applicability and Methodology

HIPtool leverages the disease burden framework of the Institute for Health Metrics Evaluation's (IHME) Global Burden of Disease (GBD) estimates, as well as the interventions framework of DCP (specifically the EUHC package), and allows tailoring to specific country needs and data. These studies represent a synthesis of global evidence on the priorities for disease burden and disease control. The following sections provide technical details on how HIPtool can be used as a preliminary step in exploring the implications of different HBP choices.

Aims and Scope

HIPtool is designed to inform discussions on priority setting and HBP design for countries at various stages of progress toward UHC. For countries defining a HBP for the first time, as well as for countries that are reviewing their HBPs, HIPtool facilitates a multi-variate approach to decision-making by incorporating available evidence on costing, impact, and disease burden within a single analytical framework.

HIPtool allows countries to estimate a HBP's potential impact and facilitate preliminary discussions on priority setting and how to improve a package by balancing its projected health impacts with equity and financial risk protection for certain populations. In addition, HIPtool can be useful for Ministries of Health seeking to draft an economic and social case to justify the need and potential returns from a national health insurance scheme or an increase in funding allocated in a certain way.

Questions HIPtool is able to address are as follows:

- What is the cost and impact of an optimised national package of health services or interventions based on global and local evidence
- What packages of health services or interventions should be prioritized for consideration for inclusion in an optimized HBP?
- What health services or interventions outside of the optimal HBP would be cost-effective and important to deliver?
- How do changes in available funding affect the interventions included in an optimal HBP?

HIPtool is primarily aimed as a gateway to designing a HBP, by comparing different possibilities of optimal HBPs depending on policy objectives and available budgets. By synthesising and linking available evidence, HIPtool seeks to provide a starting point for different options for HBPs and their potential impacts. HIPtool therefore provides an accessible starting point, preceding analysis provided by more detailed and specific costing and implementation tools such as the One Health Tool.

Data Input Requirements

HIPtool is based on country-specific disease burden data and users can select their country from a drop-down menu. This is used to pre-populate disease burden data. Default disease burden data for each primary cause is based on the IHME GBD database, although users are able to add, remove, or edit causes and associated DALYs. Causes of disability and/or mortality (e.g. HIV, Tuberculosis or Diabetes) are defined by the following properties:

1. Primary cause name
2. Health category
3. Population prevalence by year*
4. Number of people affected by year*
5. Total DALYs by year
6. Total mortality by year

** If one of these quantities is entered or updated, the other will be automatically calculated.*

All cause data is visualisable and editable, with import/export options to Excel available.

The full set of interventions included in HIPtool is based on the EUHC package published in DCP3 (Annex 3F, Volume 9). Each intervention is defined by the following properties:

1. Intervention name
2. Targeted disease(s)
3. Delivery platform
4. Unit cost per person covered
5. DALYs averted per person covered*
6. Cost per DALY averted*
7. Default coverage of intervention†
8. Maximum coverage of intervention†
9. Equity score
10. Financial risk protection score

** If one of these quantities is entered or updated, the other will be automatically calculated.*
† Default data are not present. Percentage coverage estimates are usually collated from secondary sources, and where unavailable DCP3 coverage assumptions are used.

Where possible, country-specific estimates for each of these measures are utilised; a simple tabular graphical interface is provided to easily update estimates, with full data import/export available via Excel. Default values for indicators are based on international estimates, including DCP3, and in a format that could be populated using data from sources such as Tufts Cost-Effectiveness Analysis Registry and WHO-CHOICE. All default values are visible to the user, editable, and fully documented along with assumptions in the excel template that accompanies HIPtool. In addition, the user has the option to add, edit, or delete interventions, providing a fully customisable list of interventions for a given country context.

Impact Model

Each intervention in HIPtool is linked one or more causes of disease burden classified in the GBD conducted by IHME. The linking of interventions to GBD causes of disease was carried out with guidance from WHO experts. In turn, the burden of disease data (prevalence, mortality and DALYs) associated with EUHC interventions in HIPtool is based on this linking exercise. The impact or *outcome, O,* of a given set of interventions on burden of disease is defined as:

$$O = S/(ICER/Q)$$

where O is the outcome expressed as burden averted in DALYs, S is the total amount of spending on an intervention, and Q is the quality factor ($0 < Q < 1$) that reflects realistic implementation of interventions (a 70% reduction in cost-effectiveness is the default assumption).

Existing Impact and Maximum Potential Impact

Each intervention is defined by a maximum potential impact MPI to reflect real constraints of scaling up an intervention by parameterising the upper-bounds of intervention spending for the optimisation process. Maximum potential impact is defined as the ratio between target nominal coverage T_c and current nominal coverage N_c, multiplied by the existing impact EI:

$$MPI = (T_c/N_c)EI$$

The existing impact EI is dependent on the disease burden D_B (in terms of DALYs) and the outcome of the single intervention O expressed as burden averted (in terms of DALYs). This burden is given by the ratio of the spending needed to implement the intervention S and the incremental cost-effectiveness ratio (ICER) relative to the implementation of the intervention. This ICER can be reduced by a rate Q (as mentioned above) to account for a loss of effectiveness during implementation.

$$EI = O/D_B$$

For example, a malaria intervention that currently averts 1,000 DALYs of a total 10,000 DALYs that it addresses would have an existing impact $\boldsymbol{EI} = \mathbf{10}\%$. If current nominal coverage $\boldsymbol{N_c}$ for this intervention is 40% and target nominal coverage $\boldsymbol{T_c}$ is 80%, then its maximum potential impact $\boldsymbol{MPI} = \left(\frac{\mathbf{80}}{\mathbf{40}}\right)\mathbf{10}\%$, i.e. $\boldsymbol{MPI} = 20\%$.

Equity and Financial Risk Protection Modules

As noted above, individual interventions included in HBPs have health equity and financial risk protection scores assigned to them by default, sourced from DCP3, which can be modified by the user based on in-country needs.

The defaults health equity scoring is defined in terms of the heath-adjusted age at death (HAAD). Three general HAAD cut-offs are used to assign a high- or low-ranking equity score to a health intervention. For example, if an intervention addresses a cause for which

individuals have a HAAD of less than 40 years, the intervention receives a score of 3; interventions addressing a cause with a HAAD of more than 40-50 years receives a score of 2 while causes with a HAAD more than 50 years receives a score of 1. HIPtool provides the option to include the current life expectancy, allowing for the HAAD cut-offs to be automatically scaled up or down, tailoring the health equity scores to a given context. Where data are available, additional factors can be included in the calculation of the equity score, including socio-economic status, geographic location, or gender.

The financial risk protection module of HIPtool is based on three dimensions: (1) likelihood of impoverishment (LOI) in the absence of public financing; (2) urgency of need of the intervention; and (3) average age of death and level of disability, with a favourable weighting for interventions that address high disability causing diseases and improve the health of wage-earners.

Optimisation Module

A key aim of HIPtool is to generate an HBP within a given budget and to meet three defined objectives, which are to (1) maximise DALYS averted, (2) maximise equity, and/or (3) maximise financial risk protection.

Optimisations can be run in two different modes. *Constrained mode* is used to optimise for health impact (for which the user chooses to maximise DALYs averted), with constraints imposed on equity and financial risk (by default, the constraint is that equity and financial risk protection must stay the same or improve with the optimised package compared to baseline). *Weighted mode* instead performs a user-specified weighted optimisation over health impact, equity, and financial risk protection; default weights are 60%, 20%, and 20%, respectively, normalised with respect to maximum and minimum possible outcomes for each measure. An additional constraint that may be implemented by the user is that funding for a given intervention must remain constant (i.e., be excluded from the optimisation). If we define the funding for each intervention as a budget vector $\mathbf{B}$**,** the health outcome (DALYs averted) corresponding to this budget as $O(\mathbf{B})$ (as above), the total equity as $E(\mathbf{B})$, and the total financial risk protection as $F(\mathbf{B})$, then we have:

$$E(\mathbf{B}) = \int_{t=t_0}^{t_{max}} \sum_{i=1}^{n} e_i c_i(\mathbf{B}) p_i dt,$$

$$F(\mathbf{B}) = \int_{t=t_0}^{t_{max}} \sum_{i=1}^{n} f_i c_i(\mathbf{B}) p_i dt,$$

where e and f are the equity and financial risk protection score per person covered (as defined above or user-defined), and where coverage is shown here as a function of budget $\mathbf{B}$.

Constrained optimisation is defined as

$$\max(O(\mathbf{B}))\ \text{subject to} \begin{cases} \sum \mathbf{B} = const. \\ E(\mathbf{B}) \geq E_{min} \\ F(\mathbf{B}) \geq F_{min} \end{cases},$$

where E_{min} and F_{min} are the user-specified minimum values for equity and financial risk protection, respectively.

Weighted optimisation is defined as

$$\max\left(w_o O(\mathbf{B}) + w_e E(\mathbf{B}) + w_f F(\mathbf{B})\right) \text{ subject to } \sum\mathbf{B} = const. \ ,$$

where w_o, w_e, and w_f are user-chosen weights for disease outcome, equity, and financial risk protection, respectively.
