# Supplementary material for: Using allocative efficiency analysis to inform health benefits package design for progressing towards Universal Health Coverage: Proof-of-concept studies in countries seeking decision support
Source: PLoS One. 2021 Nov 29;16(11):e0260247. doi: 10.1371/journal.pone.0260247 (PMC8629222; doi:10.1371/journal.pone.0260247)
Supplement: S2 Appendix — (PDF) [file pone.0260247.s002.pdf]

S2 Appendix: HIPtool interventions by delivery platform and DCP3 care package, with respective Global Burden of Disease causes addressed

#	EUHC intervention defined in HIPtool	DCP3 care package(s)	GBD cause(s) addressed
**Delivery platform: Community**			
1	Antenatal and postpartum education on family planning	Maternal and Newborn Health	Maternal and neonatal disorders:
2	Education on handwashing and safe disposal of children's stools	Child Health	Diarrheal diseases: ; Acute hepatitis A: ; Acute hepatitis E:
3	Pneumococcus vaccination	Child Health	Pneumococcal meningitis:
4	Rotavirus vaccination	Child Health	Other intestinal infectious diseases:
5	Provision of cotrimoxazole to children born to HIV-positive mothers	Child Health	HIV/AIDS:
6	Provision of vitamin A and zinc supplementation to children according to WHO guidelines, and provision of food supplementation to women and children in food insecure households	Child Health; School-age Health; Reproductive Health; CVD	Vitamin A deficiency: ; Other nutritional deficiencies:
7	Mass social marketing of insecticide treated nets	Child Health; School-age Health; Adult Febrile Illness	Malaria: ; Dengue: ; Zika virus: ; Encephalitis: ; Yellow fever:
8	Childhood vaccination series (diphtheria, pertussis, tetanus, polio, BCG, measles, hepatitis B, Hib, rubella)	Child Health; HIV; TB; Cancer; Congenital Disorders	Diphtheria: ; Whooping cough: ; Tetanus: ; Tuberculosis: ; Measles: ; Acute hepatitis B: ; Cirrhosis and other chronic liver diseases due to hepatitis B: ; Liver cancer due to hepatitis B: ; H influenzae type B meningitis:
9	In high malaria transmission settings, indoor residual spraying (IRS) in selected areas with high transmission and entomologic data on IRS susceptibility	Child Health; Adult Febrile Illness	Malaria:
10	Education of schoolchildren on oral health	School-age Health	Oral disorders: ; Lip and oral cavity cancer:
11	Vision prescreening by teachers; vision tests and provision of ready-made glasses on-site by eye specialists	School-age Health	Blindness and vision impairment:
12	Counseling of mothers on providing thermal care for preterm newborns (delayed bath and skin-to-skin contact)	Maternal and Newborn Health	Neonatal preterm birth:
13	School based HPV vaccination for girls	School-age Health; Reproductive Health; HIV	Cervical cancer:
14	Mass drug administration for lymphatic filariasis, onchocerciasis, schistosomiasis, soil-transmitted helminthiases and trachoma, and foodborne trematode infections	School-age Health; NTDs	Lymphatic filariasis: ; Onchocerciasis: ; Schistosomiasis: ; Trachoma: ; Food-borne trematodiases:
15	School-based education on sexual health, nutrition, and healthy lifestyle	Adolescent Health; Reproductive Health; HIV	HIV/AIDS and sexually transmitted infections: ; Acute hepatitis: ; Nutritional deficiencies: ; Cardiovascular diseases: ; Eating disorders: ; Musculoskeletal disorders: ; Neoplasms: ; Digestive diseases: ; Asthma: ; Chronic obstructive pulmonary disease: ; Substance use disorders: ; Diabetes and kidney diseases:
16	Adolescent-friendly health services including: provision of condoms to prevent STIs; provision of reversible contraception; treatment of injury in general and abuse in particular; and screening and treatment for STIs	Adolescent Health; HIV	HIV/AIDS and sexually transmitted infections: ; Acute hepatitis: ; Nutritional deficiencies: ; Cardiovascular diseases: ; Eating disorders: ; Musculoskeletal disorders: ; Neoplasms: ; Digestive diseases: ; Asthma: ; Chronic obstructive pulmonary disease:
17	Life skills training in schools to build social and emotional competencies	Adolescent Health; Mental Health	Depressive disorders: ; Anxiety disorders: ; Conduct disorder: ; Eating disorders: ; Attention-deficit/hyperactivity disorder: ; Autism spectrum disorders: ; Idiopathic developmental intellectual disability:
18	Education campaigns for the prevention of gender-based violence	Reproductive Health	Interpersonal violence:
19	In countries where it is a public health concern, prevention of FGM (may be for daughters of women of reproductive age)	Reproductive Health	Depressive disorders: ; Anxiety disorders: ; Sexual violence: ; Urinary tract infections:

#	EUHC intervention defined in HIPtool	DCP3 care package(s)	GBD cause(s) addressed
20	Provision of iron and folic acid supplementation to pregnant women, and provision of food or caloric supplementation to pregnant women in food insecure households	Reproductive Health; CVD	Maternal disorders:
21	Community-based HIV testing and counseling (for example, mobile units and venue-based testing), with appropriate referral or linkage to care and immediate initiation of lifelong ART	HIV	HIV/AIDS:
22	Household HIV testing and counseling in high-prevalence settings, with appropriate referral or linkage to care and immediate initiation of lifelong ART	HIV	HIV/AIDS:
23	Management of labor and delivery in low risk women by skilled attendants, including basic neonatal resuscitation following delivery	Maternal and Newborn Health	Maternal disorders: ; Neonatal preterm birth: ; Neonatal encephalopathy due to birth asphyxia and trauma: ; Neonatal sepsis and other neonatal infections: ; Other neonatal disorders:
24	Provision of condoms to key populations, including sex workers, men who have sex with men, people who inject drugs, transgender populations, and prisoners	HIV	HIV/AIDS and sexually transmitted infections:
25	Provision of harm reduction services such as safe injection equipment and opioid substitution therapy to people who inject drugs	HIV; Mental Health	Substance use disorders:
26	Routine contact tracing to identify individuals exposed to TB and link them to care	TB	Tuberculosis:
27	For malaria due to P. vivax, test for G6PD deficiency; if normal, add chloroquine or chloroquine plus 14-day course of primaquine	Adult Febrile Illness	Malaria:
28	Conduct larviciding and water-management programs in high malaria transmission areas where mosquito breeding sites can be identified and regularly targeted	Adult Febrile Illness	Malaria: ; Dengue: ; Zika virus: ; Encephalitis: ; Yellow fever:
29	In all malaria-endemic countries, diagnosis with rapid test or microscopy (including speciation) followed by treatment with ACTs (or current first-line combination)	Adult Febrile Illness	Malaria:
30	In high malaria transmission settings where rapid tests and microscopy are unavailable, presumptive treatment of febrile illness with ACTs (non-severe cases) or ACTs plus antibiotics (severe cases)	Adult Febrile Illness	Malaria:
31	In high malaria transmission settings, intermittent preventive treatment in infancy (except where seasonal malaria chemoprophylaxis is being provided)	Adult Febrile Illness	Malaria:
32	In low malaria transmission settings, addition of single low-dose primaquine to first-line treatment	Adult Febrile Illness	Malaria:
33	In low malaria transmission settings, case investigation, reactive case detection, proactive case detection (including mass screening and treatment)	Adult Febrile Illness	Malaria:
34	Promotion of breastfeeding or complementary feeding by lay health workers	Maternal and Newborn Health; Child Health	Nutritional deficiencies: ; Neonatal sepsis and other neonatal infections:
35	In the Sahel region, seasonal malaria chemoprophylaxis	Adult Febrile Illness	Malaria:
36	Mass drug administration in low malaria transmission settings (including high-risk groups in geographic or demographic clusters)	Adult Febrile Illness	Malaria:
37	Management of lymphedema	NTDs	Lymphatic filariasis:
38	Early detection and treatment of Chagas disease, human African trypanosomiasis, leprosy, and leishmaniases	NTDs	Chagas disease: ; African trypanosomiasis: ; Leprosy: ; Leishmaniasis:
39	Total Community Treatment for yaws	NTDs	Other neglected tropical diseases:
40	Identify and refer patients with high risk including pregnant women, young children, and those with underlying medical conditions	Pandemics	
41	In the context of an emerging infectious outbreak, provide advice and guidance on how to recognize early symptoms and signs and when to seek medical attention	Pandemics	Ebola: ; Zika virus: ; Other infectious diseases:
42	Exercise-based pulmonary rehabilitation for patients with obstructive lung disease	CVD	Chronic respiratory diseases:
43	Self-managed treatment of migraine	Mental Health	Migraine:

#	EUHC intervention defined in HIPtool	DCP3 care package(s)	GBD cause(s) addressed
44	Early identification of lead poisoning and counseling of families in remediation strategies for sources of environmental exposure	Injury	Poisoning by other means: ; Other hemoglobinopathies and hemolytic anemias:
45	Tetanus toxoid immunization among schoolchildren and among women attending antenatal care	Maternal and Newborn Health; School-age Health; Reproductive Health	Tetanus:
46	Parent training for high-risk families, including nurse home visitation for child maltreatment	Injury	Interpersonal violence:
47	WASH behavior change interventions, such as community-led total sanitation	Environmental Health	Enteric infections:
48	Cardiac and pulmonary rehabilitation programs	Rehabilitation	Chronic respiratory diseases: ; Rheumatic heart disease: ; Ischemic heart disease: ; Hypertensive heart disease: ; Non-rheumatic valvular heart disease: ; Cardiomyopathy and myocarditis: ; Atrial fibrillation and flutter: ; Aortic aneurysm: ; Peripheral artery disease: ; Endocarditis: ; Other cardiovascular and circulatory diseases:
49	Early childhood development rehabilitation interventions, including motor, sensory, and language stimulation	Rehabilitation	Autism spectrum disorders: ; Attention-deficit/hyperactivity disorder: ; Conduct disorder: ; Idiopathic developmental intellectual disability:
50	Functional interventions for self-care for individuals with disabilities	Rehabilitation	Injuries: ; Stroke: ; Musculoskeletal disorders: ; Parkinson's disease: ; Multiple sclerosis: ; Motor neuron disease:
51	Individualized environmental modifications (for example, adaptations to a house)	Rehabilitation	Injuries: ; Stroke: ; Musculoskeletal disorders: ; Parkinson's disease: ; Multiple sclerosis: ; Motor neuron disease:
52	Pressure area prevention and supportive seating interventions for wheelchair users	Rehabilitation	Injuries: ; Stroke: ; Musculoskeletal disorders: ; Parkinson's disease: ; Multiple sclerosis: ; Motor neuron disease:
53	Provision and training in the use of basic assistive products (such as canes, braille displays, and other aides) and compensatory strategies needed to communicate and perform activities of daily living	Rehabilitation	Blindness and vision impairment:
54	Training and retraining for disorders of speech, swallowing, communication, and cognition	Rehabilitation	Stroke: ; Parkinson's disease: ; Multiple sclerosis: ; Motor neuron disease:
55	Training, retraining, and exercise programs that address musculoskeletal injuries and disorders, including chronic low back and neck pain	Rehabilitation	Injuries: ; Musculoskeletal disorders:
56	HIV education and counseling for pregnant women, sex workers, people who inject drugs, men who have sex with men, and transgender individuals, and PLHIV and their partners	Maternal and Newborn Health; HIV	HIV/AIDS:
57	In high malaria transmission settings, intermittent preventive treatment in pregnancy	Maternal and Newborn Health; Adult Febrile Illness	Malaria:
58	Detection and management of acute severe malnutrition and referral in the presence of complications	Child Health	Protein-energy malnutrition:
59	Detection and treatment of childhood infections (iCCM), including referral if danger signs	Child Health	Malaria: ; Diarrheal diseases: ; Lower respiratory infections:
**Delivery platform: Health Center**			
60	Early detection and treatment of neonatal pneumonia with oral antibiotics	Maternal and Newborn Health	Neonatal sepsis and other neonatal infections:
61	Screening and management of diabetes in pregnancy (gestational diabetes or preexisting type II diabetes)	Maternal and Newborn Health; CVD; Congenital Disorders	Other maternal disorders:
62	Management of labor and delivery in low risk women (BEmNOC), including initial treatment of obstetric or delivery complications prior to transfer	Maternal and Newborn Health; Surgery	Maternal and neonatal disorders:
63	Detection and treatment of childhood infections with danger signs (IMCI)	Child Health	Nutritional deficiencies: ; Malaria: ; Measles: ; Lower respiratory infections: ; Upper respiratory infections: ; Diarrheal diseases:
64	Among all individuals who are known to be HIV positive, immediate ART initiation with regular monitoring of viral load for adherence and development of resistance	Child Health; HIV	HIV/AIDS:

#	EUHC intervention defined in HIPtool	DCP3 care package(s)	GBD cause(s) addressed
65	Psychological treatment for mood, anxiety, ADHD, and disruptive behavior disorders	Adolescent Health; Mental Health	Depressive disorders: ; Bipolar disorder: ; Anxiety disorders: ; Attention-deficit/hyperactivity disorder: ; Conduct disorder:
66	Management of complications following FGM	Reproductive Health	Sexual violence:
67	Post gender-based violence care, including counseling, provision of emergency contraception, and rape-response referral (medical and judicial)	Reproductive Health; HIV	HIV/AIDS and sexually transmitted infections: ; Depressive disorders: ; Anxiety disorders:
68	Syndromic management of common sexual and reproductive tract infections (for example uretheral discharge, genital ulcer, and others) according to WHO guidelines	Reproductive Health; HIV	Sexually transmitted infections excluding HIV:
69	Opportunistic screening for cervical cancer using visual inspection or HPV DNA testing and treatment of precancerous lesions with cryotherapy	Reproductive Health; HIV; Cancer; Surgery	Cervical cancer:
70	For individuals testing positive for hepatitis B and C, assessment of treatment eligibility by trained providers followed by initiation and monitoring of antiviral treatment when indicated	HIV	Acute hepatitis B: ; Acute hepatitis C: ; Cirrhosis and other chronic liver diseases due to hepatitis B: ; Cirrhosis and other chronic liver diseases due to hepatitis C: ; Liver cancer due to hepatitis B: ; Liver cancer due to hepatitis C:
71	Management of miscarriage or incomplete abortion and post abortion care	Maternal and Newborn Health	Maternal abortion and miscarriage:
72	Hepatitis B and C testing of individuals identified in the national testing policy (based on endemicity and risk level), with appropriate referral of positive individuals to trained providers	HIV	Acute hepatitis B: ; Acute hepatitis C: ; Cirrhosis and other chronic liver diseases due to hepatitis B: ; Cirrhosis and other chronic liver diseases due to hepatitis C: ; Liver cancer due to hepatitis B: ; Liver cancer due to hepatitis C:
73	Partner notification and expedited treatment for common STIs, including HIV	HIV	HIV/AIDS and sexually transmitted infections:
74	PrEP for discordant couples and others at high risk of infection such as commercial sex workers (in high prevalence settings)	HIV	HIV/AIDS:
75	Provider-initiated testing and counseling for HIV, STIs, and hepatitis, for all in contact with health system in high-prevalence settings, including prenatal care with appropriate referral or linkage to care including immediate ART initiation for those testing positive for HIV	HIV	HIV/AIDS and sexually transmitted infections: ; Acute hepatitis B: ; Acute hepatitis C: ; Cirrhosis and other chronic liver diseases due to hepatitis B: ; Cirrhosis and other chronic liver diseases due to hepatitis C: ; Liver cancer due to hepatitis B: ; Liver cancer due to hepatitis C:
76	As resources permit, hepatitis B vaccination of high-risk populations, including healthcare workers, PWID, MSM, household contacts, and persons with multiple sex partners	HIV; Cancer	Acute hepatitis B: ; Cirrhosis and other chronic liver diseases due to hepatitis B: ; Liver cancer due to hepatitis B:
77	Provision of voluntary medical male circumcision service in settings with high prevalence of HIV	HIV; Surgery	HIV/AIDS and sexually transmitted infections:
78	For PLHIV and children under five who are close contacts or household members of individuals with active TB, perform symptom screening and chest radiograph; if there is no active TB, provide isoniazid preventive therapy according to current WHO guidelines	TB	Tuberculosis:
79	Diagnosis of TB, including assessment of rifampicin resistance using rapid molecular diagnostics (UltraXpert), and initiation of first-line treatment per current WHO guidelines for drug-susceptible TB; referral for confirmation, further assessment of drug resistance, and treatment of drug-resistant TB	TB	Tuberculosis:
80	Screening for HIV in all individuals with a diagnosis of active TB; if HIV infection is present, start (or refer for) ARV treatment and HIV care	TB	HIV/AIDS:

#	EUHC intervention defined in HIPtool	DCP3 care package(s)	GBD cause(s) addressed
81	Screening for latent TB infection following a new diagnosis of HIV, followed by yearly screening among PLHIV at high risk of TB exposure; initiation of isoniazid preventive therapy among all individuals who screen positive but do not have evidence of active TB	TB	Tuberculosis:
82	Management of preterm premature rupture of membranes, including administration of antibiotics	Maternal and Newborn Health	Maternal sepsis and other maternal infections: ; Indirect maternal deaths: ; Late maternal deaths: ; Neonatal preterm birth: ; Neonatal sepsis and other neonatal infections:
83	Evaluation and management of fever in clinically stable individuals using WHO IMAI guidelines, with referral of unstable individuals to first-level hospital care	Adult Febrile Illness	Communicable, maternal, neonatal, and nutritional diseases
84	Focused use of vaccines for endemic infections, such as dengue, JEV, typhoid, meningococcus, and others	Adult Febrile Illness	Typhoid and paratyphoid: ; Dengue: ; Yellow fever: ; Encephalitis:
85	Provision of insecticide-treated nets to children and pregnant women attending Health Center	Adult Febrile Illness	Malaria: ; Dengue: ; Zika virus: ; Encephalitis: ; Yellow fever:
86	Identify and refer to higher levels of health care patients with signs of progressive illness	Pandemics	
87	Stockpile and consider treating early high risk patients with antiviral medications according to nationally endorsed guidelines	Pandemics	
88	Annual flu vaccination and pneumococcal vaccine every five years for individuals with underlying lung disease	CVD	Pneumococcal meningitis:
89	Long-term combination therapy for persons with multiple CVD risk factors, including screening for CVD in community settings using non-lab-based tools to assess overall CVD risk	CVD	Cardiovascular diseases: ; Chronic respiratory diseases:
90	Low-dose inhaled corticosteroids and bronchodilators for asthma and for selected patients with COPD	CVD	Chronic obstructive pulmonary disease: ; Asthma:
91	Provision of aspirin for all cases of suspected acute myocardial infarction	CVD	Ischemic heart disease:
92	Screening and management of albuminuric kidney disease with ACEi or ARBs, including targeted screening among people with diabetes	CVD	Chronic kidney disease:
93	Provision of condoms and hormonal contraceptives, including emergency contraceptives	Maternal and Newborn Health	HIV/AIDS and sexually transmitted infections:
94	Screening and management of diabetes among at-risk adults, including glycemic control, management of blood pressure and lipids, and consistent foot care	CVD	Diabetes mellitus:
95	Secondary prophylaxis with penicillin for rheumatic fever or established rheumatic heart disease	CVD	Rheumatic heart disease:
96	Treatment of acute pharyngitis in children to prevent rheumatic fever	CVD	Rheumatic heart disease:
97	Long term management of ischemic heart disease, stroke, and peripheral vascular disease with aspirin, beta blockers, ACEi, and statins (as indicated) to reduce risk of further events	CVD	Cardiovascular diseases: ; Chronic kidney disease:
98	Medical management of heart failure with diuretics, beta-blockers, ACEi, and mineralocorticoid antagonists	CVD	Cardiovascular diseases: ; Chronic kidney disease:
99	Opportunistic screening for hypertension for all adults and initiation of treatment among individuals with severe hypertension and/or multiple risk factors	CVD	Cardiovascular diseases: ; Chronic kidney disease:
100	Tobacco cessation counseling, and use of nicotine replacement therapy in certain circumstances	CVD; Cancer	Cardiovascular diseases: ; Lip and oral cavity cancer: ; Nasopharynx cancer: ; Other pharynx cancer: ; Esophageal cancer: ; Stomach cancer: ; Pancreatic cancer: ; Larynx cancer: ; Tracheal, bronchus, and lung cancer: ; Cervical cancer: ; Acute myeloid leukemia:
101	Essential palliative care and pain control measures, including oral immediate release morphine and medicines for associated symptoms	Cancer; Palliative Care	
102	Interventions to support caregivers of patients with dementia	Mental Health	Depressive disorders: ; Anxiety disorders:

#	EUHC intervention defined in HIPtool	DCP3 care package(s)	GBD cause(s) addressed
103	Management of bipolar disorder using generic mood-stabilizing medications and psychosocial treatment	Mental Health	Bipolar disorder:
104	Counseling of mothers on providing kangaroo care for newborns	Maternal and Newborn Health	Other neonatal disorders:
105	Management of depression and anxiety disorders with psychological and generic antidepressant therapy	Mental Health	Depressive disorders: ; Anxiety disorders:
106	Management of epilepsy, including acute stabilization and long-term management with generic anti-epileptics	Mental Health	Epilepsy:
107	Management of schizophrenia using generic anti-psychotic medications and psychosocial treatment	Mental Health	Schizophrenia:
108	Screening and brief intervention for alcohol use disorders	Mental Health; Injury	Alcohol use disorders: ; Liver cancer due to alcohol use: ; Alcoholic cardiomyopathy: ; Cirrhosis and other chronic liver diseases due to alcohol use:
109	Exercise programs for upper extremity injuries and disorders	Musculoskeletal	Transport injuries: ; Unintentional injuries: ; Exposure to forces of nature: ; Other unintentional injuries: ; Exposure to mechanical forces: ; Foreign body: ; Self-harm and interpersonal violence: ; Other musculoskeletal disorders:
110	Calcium and vitamin D supplementation for primary prevention of osteoporosis in high-risk individuals	Musculoskeletal	Other musculoskeletal disorders:
111	Targeted screening for congenital hearing loss in high-risk children using otoacoustic emissions testing	Congenital Disorders	Age-related and other hearing loss:
112	Dental extraction	Surgery	Caries of deciduous teeth: ; Caries of permanent teeth: ; Periodontal diseases:
113	Drainage of dental abscess	Surgery	Caries of deciduous teeth: ; Caries of permanent teeth:
114	Drainage of superficial abscess	Surgery	Bacterial skin diseases:
115	Management of neonatal sepsis, pneumonia, and meningitis using injectable and oral antibiotics	Maternal and Newborn Health	Neonatal sepsis and other neonatal infections:
116	Management of non-displaced fractures	Surgery	Transport injuries: ; Unintentional injuries: ; Exposure to forces of nature: ; Other unintentional injuries: ; Exposure to mechanical forces: ; Foreign body: ; Self-harm and interpersonal violence:
117	Resuscitation with basic life support measures	Surgery	Injuries:
118	Suturing laceration	Surgery	Transport injuries: ; Unintentional injuries: ; Exposure to forces of nature: ; Other unintentional injuries: ; Exposure to mechanical forces: ; Foreign body: ; Self-harm and interpersonal violence:
119	Treatment of caries	Surgery	Caries of deciduous teeth: ; Caries of permanent teeth:
120	Basic management of musculoskeletal and neurological injuries and disorders, such as prescription of simple exercises and sling or cast provision	Rehabilitation	Musculoskeletal disorders: ; Transport injuries: ; Unintentional injuries: ; Exposure to forces of nature: ; Other unintentional injuries: ; Exposure to mechanical forces: ; Foreign body: ; Self-harm and interpersonal violence:
121	Review of prosthetics, orthotics, and splints, with referral to hospital if indicated	Rehabilitation	Transport injuries: ; Unintentional injuries: ; Exposure to forces of nature: ; Other unintentional injuries: ; Exposure to mechanical forces: ; Foreign body: ; Self-harm and interpersonal violence:
122	Psychosocial support and counseling services for individuals with serious, complex, or life-limiting health problems and their caregivers	Palliative Care	Depressive disorders: ; Anxiety disorders:
123	Expanded palliative care and pain control measures, including prevention and relief of all physical and psychological symptoms of suffering	Palliative Care	
124	Health center pathology services	Pathology	
125	Pharmacological termination of pregnancy	Maternal and Newborn Health	Maternal abortion and miscarriage:
126	PMTCT of HIV (Option B+) and syphilis	Maternal and Newborn Health; HIV	HIV/AIDS: ; Syphilis:

#	EUHC intervention defined in HIPtool	DCP3 care package(s)	GBD cause(s) addressed
127	Screening and management of hypertensive disorders in pregnancy	Maternal and Newborn Health	Maternal hypertensive disorders:
**Delivery platform: First-level Hospital**			
128	Detection and management of fetal growth restriction	Maternal and Newborn Health	Protein-energy malnutrition: ; Other maternal disorders: ; Other neonatal disorders:
129	Surgical termination of pregnancy by manual vacuum aspiration and dilation and curettage	Maternal and Newborn Health; Surgery	Maternal abortion and miscarriage:
130	Full supportive care for severe childhood infections with danger signs	Child Health	Lower respiratory infections: ; Urinary tract infections: ; Diarrheal diseases: ; Malaria: ; HIV/AIDS: ; Drug-susceptible tuberculosis: ; Multidrug-resistant tuberculosis without extensive drug resistance: ; Extensively drug-resistant tuberculosis: ; Zika virus: ; Dengue: ; Ebola:
131	Management of severe acute malnutrition associated with serious infection	Child Health	Protein-energy malnutrition:
132	Early detection and treatment of early-stage cervical cancer	Reproductive Health; HIV	Cervical cancer:
133	Insertion and removal of long-lasting contraceptives	Reproductive Health; Surgery	Maternal abortion and miscarriage: ; Indirect maternal deaths: ; Late maternal deaths: ; Ectopic pregnancy:
134	Tubal ligation	Reproductive Health; Surgery	Maternal abortion and miscarriage: ; Indirect maternal deaths: ; Late maternal deaths: ; Ectopic pregnancy:
135	Vasectomy	Reproductive Health; Surgery	Maternal abortion and miscarriage: ; Indirect maternal deaths: ; Late maternal deaths: ; Ectopic pregnancy:
136	Referral of cases of treatment failure for drug susceptibility testing; enrollment of those with MDR-TB for treatment per WHO guidelines (either short or long regimen)	TB	Multidrug-resistant tuberculosis without extensive drug resistance: ; HIV/AIDS - Multidrug-resistant Tuberculosis without extensive drug resistance:
137	Evaluation and management of fever in clinically unstable individuals using WHO IMAI guidelines, including empiric parenteral antimicrobials and antimalarials and resuscitative measures for septic shock	Adult Febrile Illness	Communicable, maternal, neonatal, and nutritional diseases:
138	Management of severe malaria, including early detection and provision of rectal artesunate in community settings followed by parenteral artesunate and full-course of ACT	Adult Febrile Illness	Malaria:
139	Induction of labor post-term	Maternal and Newborn Health	Maternal hemorrhage: ; Other maternal disorders: ; Maternal sepsis and other maternal infections: ; Maternal hypertensive disorders: ; Maternal obstructed labor and uterine rupture: ; Maternal abortion and miscarriage: ; Ectopic pregnancy: ; Indirect maternal deaths: ; Late maternal deaths: ; Neonatal encephalopathy due to birth asphyxia and trauma:
140	Management of acute coronary syndromes with aspirin, unfractionated heparin, and generic thrombolytics (when indicated)	CVD	Ischemic heart disease:
141	Management of acute critical limb ischemia with unfractionated heparin and revascularization where available, with amputation as a last resort	CVD	Cardiovascular diseases:
142	Management of acute exacerbations of asthma and COPD using systemic steroids, inhaled beta-agonists, and, if indicated, oral antibiotics and oxygen therapy	CVD	Chronic obstructive pulmonary disease: ; Asthma:
143	Medical management of acute heart failure	CVD	Other cardiovascular and circulatory diseases:
144	Management of bowel obstruction	Cancer; Surgery	Paralytic ileus and intestinal obstruction:
145	Calcium and vitamin D supplementation for secondary prevention of osteoporosis	Musculoskeletal	Other musculoskeletal disorders:

#	EUHC intervention defined in HIPtool	DCP3 care package(s)	GBD cause(s) addressed
146	Combination therapy, including low-dose corticosteroids and generic disease-modifying antirheumatic drugs (including methotrexate), for individuals with moderate to severe rheumatoid arthritis	Musculoskeletal	Rheumatoid arthritis:
147	In settings where sickle cell disease is a public health concern, universal newborn screening followed by standard prophylaxis against bacterial infections and malaria	Congenital Disorders	Sickle cell disorders: ; Neonatal sepsis and other neonatal infections: ; Malaria:
148	In settings where specific single-gene disorders are a public health concern (for example, thalassemias), retrospective identification of carriers plus prospective (premarital) screening and counseling to reduce rates of conception	Congenital Disorders	Hemoglobinopathies and hemolytic anemias:
149	Universal newborn screening for congenital endocrine or metabolic disorders (for example, congenital hypothyroidism, phenylketonuria) that have high incidence rates and for which long-term treatment is feasible in limited resource settings	Congenital Disorders	Digestive congenital anomalies: ; Urogenital congenital anomalies: ; Other congenital birth defects:
150	Jaundice management with phototherapy	Maternal and Newborn Health	Hemolytic disease and other neonatal jaundice:
151	Management of intoxication/poisoning syndromes using widely available agents; e.g., activated charcoal, naloxone, bicarbonate, antivenin	Injury	Substance use disorders: ; Poisonings: ; Venomous animal contact:
152	Appendectomy	Surgery	Appendicitis:
153	Assisted vaginal delivery using vacuum extraction or forceps	Surgery	Maternal obstructed labor and uterine rupture:
154	Burr hole to relieve acute elevated intracranial pressure	Surgery	Intracerebral hemorrhage:
155	Colostomy	Surgery	Colon and rectum cancer:
156	Escharotomy or fasciotomy	Surgery	Fire, heat, and hot substances:
157	Fracture reduction and placement of external fixator and use of traction for fractures	Surgery	Transport injuries: ; Unintentional injuries: ; Exposure to forces of nature: ; Other unintentional injuries: ; Exposure to mechanical forces: ; Foreign body: ; Self-harm and interpersonal violence:
158	Hernia repair including emergency surgery	Surgery	Inguinal, femoral, and abdominal hernia:
159	Hysterectomy for uterine rupture or intractable postpartum hemorrhage	Surgery	Maternal hemorrhage:
160	Irrigation and debridement of open fractures	Surgery	Transport injuries: ; Unintentional injuries: ; Exposure to forces of nature: ; Other unintentional injuries: ; Exposure to mechanical forces: ; Foreign body: ; Self-harm and interpersonal violence:
161	Management of eclampsia with magnesium sulfate, including initial stabilization at Health Center	Maternal and Newborn Health	Maternal hypertensive disorders:
162	Management of osteomyelitis, including surgical debridement for refractory cases	Surgery	Other musculoskeletal disorders:
163	Management of septic arthritis	Surgery	Other musculoskeletal disorders:
164	Relief of urinary obstruction by catheterization or suprapubic cystostomy	Surgery	Urinary tract infections: ; Urolithiasis: ; Benign prostatic hyperplasia: ; Other urinary diseases: ; Cervical cancer: ; Bladder cancer:
165	Removal of gallbladder including emergency surgery	Surgery	Gallbladder and biliary diseases: ; Gallbladder and biliary tract cancer:
166	Repair of perforations (for example, perforated peptic ulcer, typhoid ileal perforation)	Surgery	Peptic ulcer disease: ; Other intestinal infectious diseases: ; Paralytic ileus and intestinal obstruction:
167	Resuscitation with advanced life support measures, including surgical airway	Surgery	Injuries:
168	Basic skin grafting	Surgery	Fire, heat, and hot substances: ; Malignant skin melanoma: ; Decubitus ulcer:
169	Surgery for filarial hydrocele	Surgery	Lymphatic filariasis:

#	EUHC intervention defined in HIPtool	DCP3 care package(s)	GBD cause(s) addressed
170	Trauma laparotomy	Surgery	Transport injuries: ; Unintentional injuries: ; Exposure to forces of nature: ; Other unintentional injuries: ; Exposure to mechanical forces: ; Foreign body: ; Self-harm and interpersonal violence:
171	Trauma-related amputations	Surgery	Transport injuries: ; Unintentional injuries: ; Exposure to forces of nature: ; Other unintentional injuries: ; Exposure to mechanical forces: ; Foreign body: ; Self-harm and interpersonal violence:
172	Management of maternal sepsis, including early detection at Health Center	Maternal and Newborn Health	Maternal sepsis and other maternal infections:
173	Tube thoracostomy	Surgery	Tracheal, bronchus, and lung cancer: ; Transport injuries: ; Unintentional injuries: ; Exposure to forces of nature: ; Other unintentional injuries: ; Exposure to mechanical forces: ; Foreign body: ; Self-harm and interpersonal violence:
174	Assessment, provision and training in the use of assistive products, including assistive devices for hearing	Rehabilitation	Age-related and other hearing loss: ; Blindness and vision impairment:
175	Compression therapy for amputations, burns, and vascular or lymphatic disorders	Rehabilitation	Fire, heat, and hot substances: ; Lymphatic filariasis:
176	Evaluation and acute management of swallowing dysfunction	Rehabilitation	Stroke: ; Lip and oral cavity cancer: ; Nasopharynx cancer: ; Other pharynx cancer: ; Esophageal cancer:
177	Fabrication, fitting, and training in the use of prosthetics, orthotics, and splints	Rehabilitation	Transport injuries: ; Unintentional injuries: ; Exposure to forces of nature: ; Other unintentional injuries: ; Exposure to mechanical forces: ; Foreign body: ; Self-harm and interpersonal violence:
178	Initial assessment, and prescription, and provision of individualized interventions for musculoskeletal, cardiopulmonary, neurological, speech and communication, and cognitive deficits, including training in preparation for discharge	Rehabilitation	Musculoskeletal disorders: ; Neurological disorders: ; Chronic respiratory diseases: ; Cardiovascular diseases:
179	Mobilization activities following acute injury or illness	Rehabilitation	Cardiovascular diseases: ; Chronic respiratory diseases: ; Transport injuries: ; Unintentional injuries: ; Exposure to forces of nature: ; Other unintentional injuries: ; Exposure to mechanical forces: ; Foreign body: ; Self-harm and interpersonal violence:
180	Prevention and relief of refractory suffering and of acute pain related to surgery, serious injury, or other serious, complex or life-limiting health problems	Palliative Care	
181	First-level hospital pathology services	Pathology	
182	Management of newborn complications, neonatal meningitis, and other very serious infections requiring continuous supportive care (IV fluids, oxygen, etc.)	Maternal and Newborn Health	Neonatal sepsis and other neonatal infections:
183	Management of preterm labor with corticosteroids, including early detection at Health Center	Maternal and Newborn Health	Neonatal preterm birth:
184	Management of labor and delivery in high risk women, including operative delivery (CEmNOC)	Maternal and Newborn Health; Surgery	Maternal and neonatal disorders:
185	Surgery for ectopic pregnancy	Maternal and Newborn Health; Surgery	Ectopic pregnancy:
**Delivery platform: Referral and Specialty Hospital**			
186	Full supportive care for preterm newborns	Maternal and Newborn Health	Neonatal disorders:
187	Elective surgical repair of common orthopedic injuries (for example, meniscal and ligamentous tears) in individuals with severe functional limitation	Musculoskeletal	Transport injuries: ; Unintentional injuries: ; Exposure to forces of nature: ; Other unintentional injuries: ; Exposure to mechanical forces: ; Foreign body: ; Self-harm and interpersonal violence:

#	EUHC intervention defined in HIPtool	DCP3 care package(s)	GBD cause(s) addressed
188	Urgent, definitive surgical management of orthopedic injuries (for example, by open reduction and internal fixation)	Musculoskeletal	Transport injuries: ; Unintentional injuries: ; Exposure to forces of nature: ; Other unintentional injuries: ; Exposure to mechanical forces: ; Foreign body: ; Self-harm and interpersonal violence:
189	Repair of cleft lip and cleft palate	Congenital Disorders; Surgery	Orofacial clefts:
190	Repair of club foot	Congenital Disorders; Surgery	Congenital musculoskeletal and limb anomalies:
191	Cataract extraction and insertion of intraocular lens	Surgery	Cataract:
192	Repair of anorectal malformations and Hirschsprung's Disease	Surgery	Digestive congenital anomalies:
193	Repair of obstetric fistula	Surgery	Other maternal disorders: ; Maternal obstructed labor and uterine rupture: ; Late maternal deaths:
194	Insertion of shunt for hydrocephalus	Surgery	Other neurological disorders:
195	Surgery for trachomatous trichiasis	Surgery	Chlamydial infection: ; Trachoma: ; Other vision loss:
196	Referral-level hospital pathology services	Pathology	
197	Specialized TB services, including management of MDR- and XDR-TB treatment failure and surgery for TB	TB	Tuberculosis:
198	Specialty pathology services	Pathology	
199	Management of refractory febrile illness including etiologic diagnosis at reference microbiological laboratory	Adult Febrile Illness	Communicable, maternal, neonatal, and nutritional diseases:
200	Management of acute ventilatory failure due to acute exacerbations of asthma and COPD; in COPD use of bilevel positive airway pressure preferred	CVD	Chronic obstructive pulmonary disease: ; Asthma:
201	Retinopathy screening via telemedicine, followed by treatment using laser photocoagulation	CVD	Diabetes mellitus: ; Hypertensive heart disease: ; Age-related macular degeneration: ; Other vision loss:
202	Use of percutaneous coronary intervention for acute myocardial infarction where resources permit	CVD	Ischemic heart disease:
203	Treatment of early stage breast cancer with appropriate multimodal approaches (including generic chemotherapy), with curative intent, for cases that are detected by clinical examination at Health Center	Cancer	Breast cancer:
204	Treatment of early stage colorectal cancer with appropriate multimodal approaches (including generic chemotherapy), with curative intent, for cases that are detected by clinical examination at Health Center	Cancer	Colon and rectum cancer:
205	Treatment of early-stage childhood cancers (such as Burkitt and Hodgkin lymphoma, acute lymphoblastic leukemia, retinoblastoma, and Wilms tumor) with curative intent in pediatric cancer units or hospitals	Cancer	Brain and nervous system cancer: ; Hodgkin lymphoma: ; Acute lymphoid leukemia: ; Other leukemia: ; Kidney cancer: ; Other malignant neoplasms:
**Population-based Health Interventions**			
206	Mass media messages concerning sexual and reproductive health and mental health for adolescents	Adolescent Health; HIV	HIV/AIDS and sexually transmitted infections: ; Depressive disorders: ; Bipolar disorder: ; Anxiety disorders: ; Eating disorders: ; Attention-deficit/hyperactivity disorder: ; Conduct disorder: ; Substance use disorders:
207	Develop and implement a plan to ensure surge capacity in hospital beds, stockpiles of disinfectants, equipment for supportive care, and personal protective equipment	Pandemics	
208	Develop plans and legal standards for curtailing interactions between infected persons and uninfected population and implement and evaluate infection control measures in health facilities	Pandemics	
209	Ensure influenza vaccine security at national and subnational level	Pandemics	
210	Mass media messages concerning awareness on handwashing and health effects of household air pollution	Environmental Health	Diarrheal diseases: ; Acute hepatitis A: ; Acute hepatitis E: ; Chronic respiratory diseases:

#	EUHC intervention defined in HIPtool	DCP3 care package(s)	GBD cause(s) addressed
211	Mass media messages concerning healthy eating or physical activity	Adolescent Health; HIV; CVD; Musculoskeletal	Nutritional deficiencies: ; Cardiovascular diseases: ; Eating disorders: ; Musculoskeletal disorders: ; Neoplasms: ; Digestive diseases: ; Asthma: ; Chronic obstructive pulmonary disease: ; Substance use disorders: ; Diabetes and kidney diseases:
212	Mass media messages concerning use of tobacco and alcohol	Adolescent Health; HIV; CVD; Musculoskeletal	Cardiovascular diseases: ; Lip and oral cavity cancer: ; Nasopharynx cancer: ; Other pharynx cancer: ; Esophageal cancer: ; Stomach cancer: ; Pancreatic cancer: ; Larynx cancer: ; Tracheal, bronchus, and lung cancer: ; Cervical cancer: ; Acute myeloid leukemia: ; Alcohol use disorders: ; Liver cancer due to alcohol use: ; Alcoholic cardiomyopathy: ; Cirrhosis and other chronic liver diseases due to alcohol use:
213	Mass media encouraging use of condoms, voluntary medical male circumcision, and STI testing	HIV	HIV/AIDS and sexually transmitted infections:
214	Systematic identification of individuals with TB symptoms among high-risk groups and linkage to care ("active case finding")	TB	Tuberculosis:
215	Sustained vector management for Chagas disease, visceral leishmaniasis, dengue, and other nationally important causes of nonmalarial fever	Adult Febrile Illness; NTDs; CVD	Chagas disease: ; Dengue: ; Visceral leishmaniasis: ; Typhoid and paratyphoid: ; Yellow fever: ; Encephalitis:
216	Conduct a comprehensive assessment of International Health Regulations (IHR) competencies using the Joint External Evaluation tool and develop, cost, finance and implement an action plan to address gaps in preparedness and response	Pandemics	
217	Conduct simulation exercises and health worker training for outbreak events including outbreak investigation, contact tracing and emergency response	Pandemics	
218	Decentralize stocks of antiviral medications in order to reach at-risk groups and disadvantaged populations	Pandemics	
