# Supplementary material for: Using allocative efficiency analysis to inform health benefits package design for progressing towards Universal Health Coverage: Proof-of-concept studies in countries seeking decision support
Source: PLoS One. 2021 Nov 29;16(11):e0260247. doi: 10.1371/journal.pone.0260247 (PMC8629222; doi:10.1371/journal.pone.0260247)
Supplement: S3 Appendix — (PDF) [file pone.0260247.s003.pdf]

S3 Appendix: Local equity and financial risk protection scores (Armenia)

The equity scores (1 – 3) were defined to reflect social groups identified as priority for coverage in the Armenian health system. The highest score (3) was assigned to Armenia UHC interventions that targeted pregnant and lactating mothers, children and disabled populations, and individuals suffering from poverty-related diseases such as malnutrition and TB. A score of 2 was assigned to AUHC interventions for social groups that may experience stigma, such as the review of and referral for prosthetics, orthotics, and splints, while AUHC interventions targeting the general population targets received the lowest score (1). Two independent reviewers agreed on the final scores.

The financial risk protection scores (1 – 6) were based on the likelihood of impoverishment in the absence of public financing for an AUHC intervention. They were composed of 1-5 points linked to the local unit price, and an additional 1 point if expenditure was likely recurrent beyond one year due to the chronicity of disease. The unit price bands were as follows In Armenia Dram): Band 1: <3,000, Band 2: 3,000-12,000, Band 3: 12,001-50,000, Band 4: 50,001-180,000, Band 5: 180,001+.

Examples:

**EUHC code**	**AHIP intervention**	**Unit price (Armenia Dram)**	**FRP score for price band (1-5)**	**FRP score for chronic (=1)**	**FRP score (1-6)**	**Equity score (1-3)**
C1	Perinatal care (including consultation on family planning, breasfeeding, skin-to-skin contact, etc.)	29,318	3		**3**	**3**
C11	Pneumococcus vaccination	10,605	2		**2**	**3**
C12	Rotavirus vaccination	5,808	2		**2**	**3**
C14	Micronutrient supplementation to children as clinically [illegible]	2,410	1	1	**2**	**3**
C16	Childhood vaccination series (diptheria, pertussis, tetanus, polio, BCG, measles, hepatitis B, Hib,MMR)	44,881	3		**3**	**3**
C18	Primary preventive dental services for children (aged 12)	1,200	1		**1**	**2**
C19	Vision screening at PHCs, prescription of glasses	701	1		**1**	**2**
C20	School based HPV vaccination for girls	8,372	2		**2**	**2**
C27	Provision of drugs for free to pregnant women at preferential conditions by OBGYN	6,457	2		**2**	**3**
C28 (incl. C29, HC28)	Community-based HIV testing	2,065	1		**1**	**2**
C3 (incl. FLH3, HC11)	Management of labor and delivery in low risk women including basic neonatal resuscitation following delivery and	164,124	4		**4**	**3**
C30	Provision of condoms to key populations, including sex workers, men who have sex with men, people who inject drugs, transgender populations, and prisoners	3,108	2	1	**3**	**2**
C31	Provision of harm reduction services such as safe injection equipment and opioid substitution therapy to people who inject drugs, and Medical Care in Yerevan Narcology Dispensary	57,366	4	1	**5**	**3**
C45	Inpatient examination of primary patients to clarify the diagnosis (in infectious services)	23,071	3		**3**	**2**
