# Supplementary material for: Using allocative efficiency analysis to inform health benefits package design for progressing towards Universal Health Coverage: Proof-of-concept studies in countries seeking decision support
Source: PLoS One. 2021 Nov 29;16(11):e0260247. doi: 10.1371/journal.pone.0260247 (PMC8629222; doi:10.1371/journal.pone.0260247)

S4 Appendix: Additional optimization results

Côte d’Ivoire

Figure S1. Current and optimized DALY impact by national disease program

Disease program results: Total averted DALYs increase by 22% under the optimized scenario, particularly for health promotion, surgery and NCDs. Optimizing the intervention mix within programs results in higher DALYs averted by all disease programs.

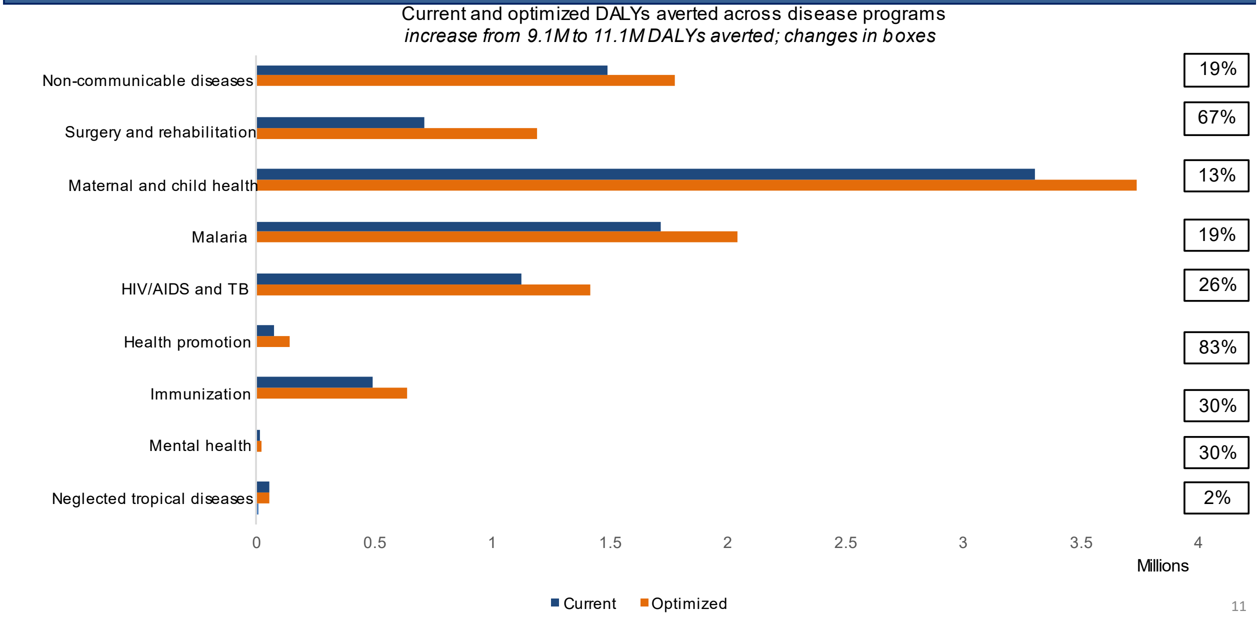

Figure S2. Interventions with the largest spending increases and DALY impact upon optimization

Changes in intervention spending and DALYs averted: Scale up of interventions targeting heart disease, surgery needs and maternal and child health is associated with significant increases in DALYs averted.

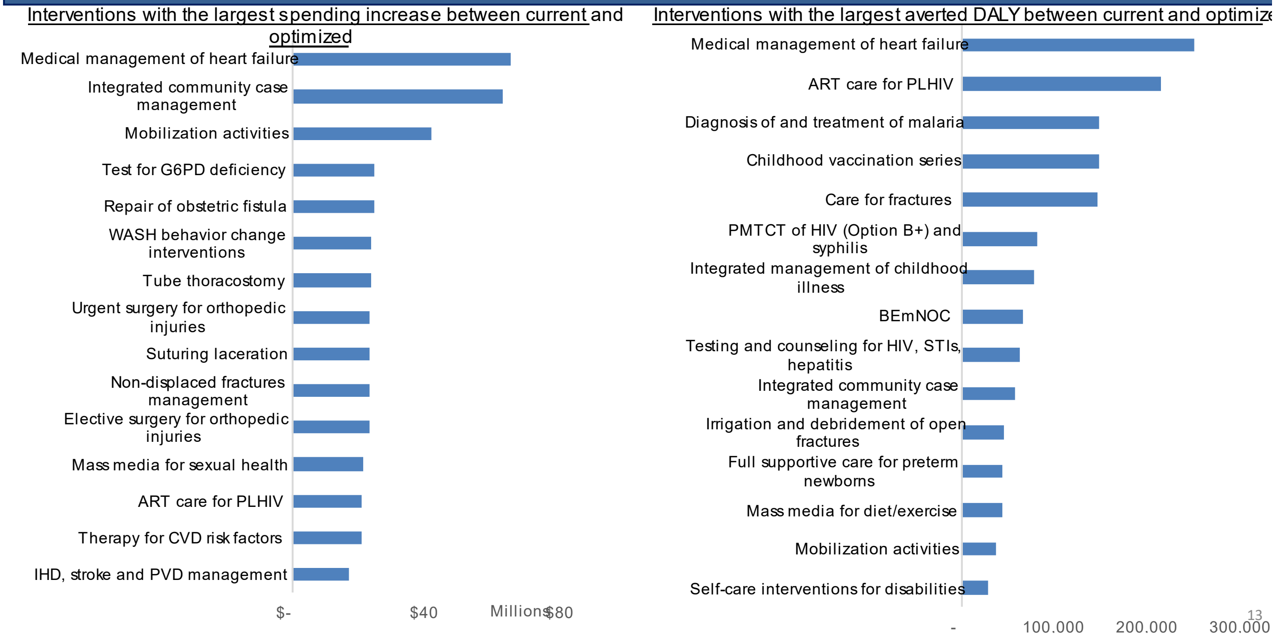

Zimbabwe

Figure S3. Current and optimized spending and impact by health delivery platform

Note: Non-optimized interventions were part of the spending envelope but because these interventions cannot be linked to a specific disease burden, they do not have an estimate of DALY impact.

A) Estimated 2016 actual and optimized spending

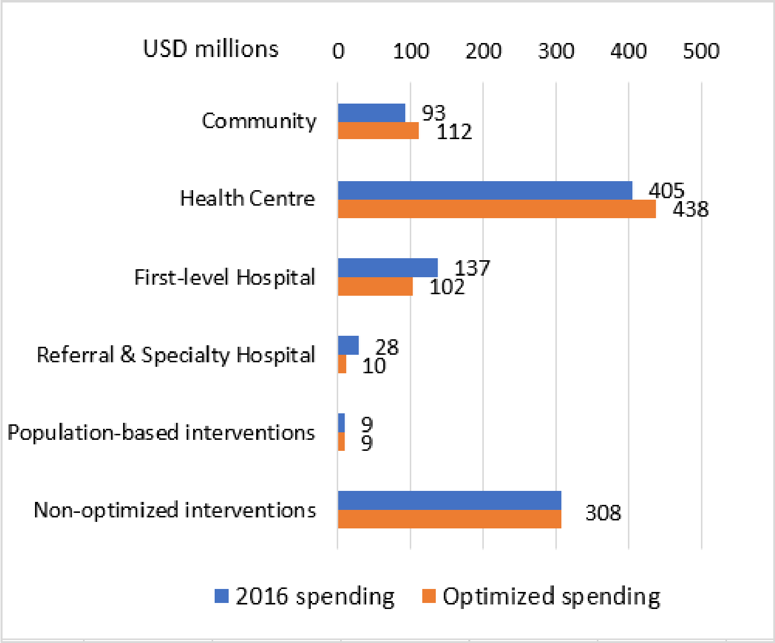

B) Estimated DALY impacts

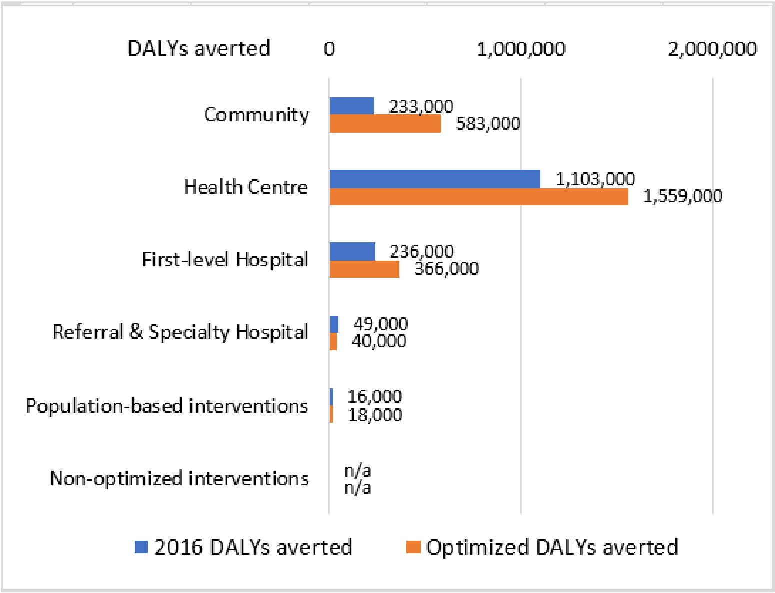

Figure S4. Current and optimized spending and impact by national disease program

Note: Spending for interventions not included in the optimization is not shown.

A) Estimated 2016 actual and optimized spending

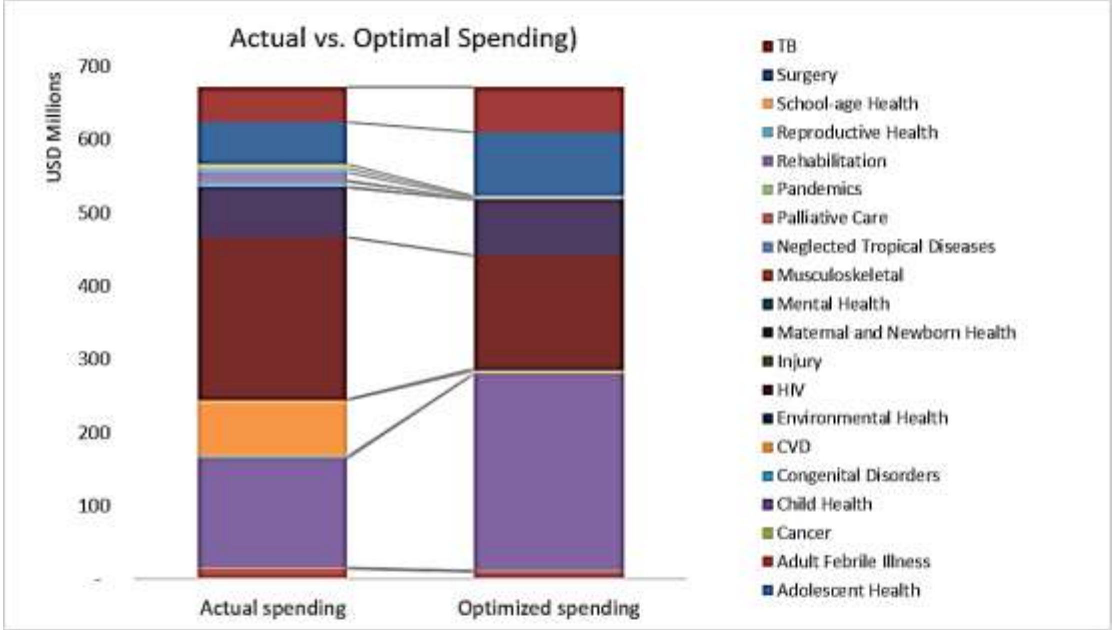

B) Estimated DALY impacts

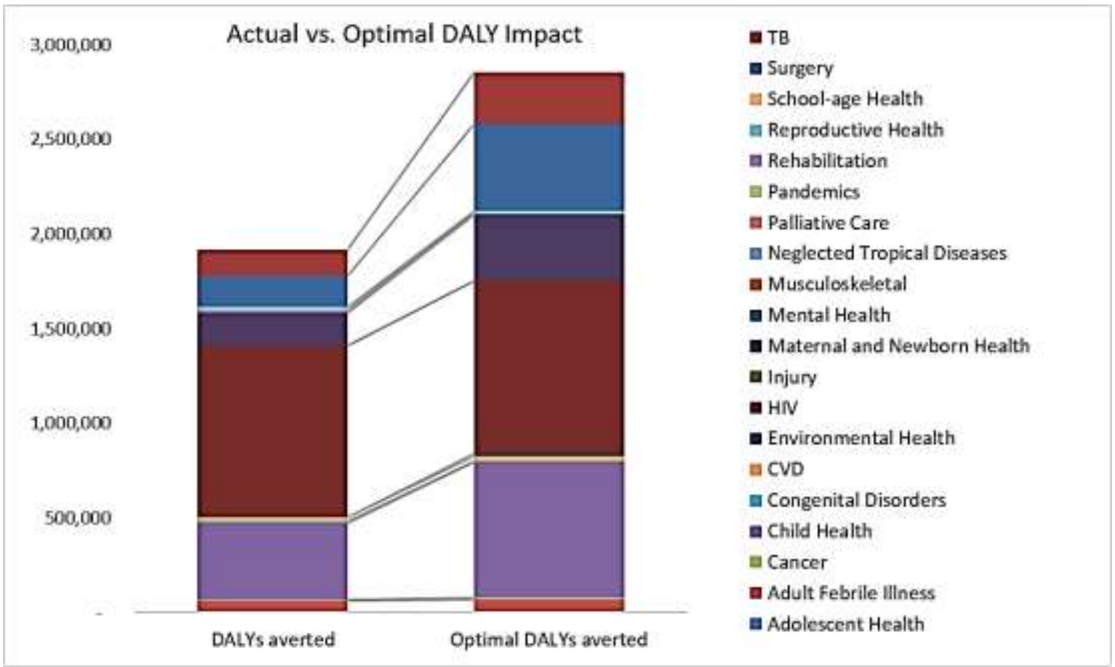

Supplement: S4 Appendix — (PDF) [file pone.0260247.s004.pdf]
